# “I prefer a male nurse to a female nurse”: patients’ preference for, and satisfaction with nursing care provided by male nurses at the Komfo Anokye teaching hospital

**DOI:** 10.1186/s12912-019-0369-4

**Published:** 2019-10-21

**Authors:** Hayford Isaac Budu, Emmanuel Mawuli Abalo, Victoria Bubunyo Bam, Deus Osei Agyemang, Shirley Noi, Florence A. Budu, Prince Peprah

**Affiliations:** 1 Department of Nursing, Kwame Nkrumah University of Science and Technology, Kumasi, Ghana; 2 Department of Geography and Rural Development, Kwame Nkrumah University of Science and Technology, Kumasi, Ghana; 3 Saint Michael Midwifery Training School, Pramso, Ghana

**Keywords:** Male nurse, Patient, Preference, Satisfaction, Komfo Anokye teaching hospital

## Abstract

**Background:**

Although most male nurses join the profession for self-actualisation, the cultural and societal stereotyping of male nurses as “He-Man”, “gay” and “troublemakers”, and their marginalisation at the hospital during certain personal and intimate care procedures, tend to deepen the existing gender discrimination prevalent within the nursing profession. This study therefore assessed patients’ preference for, and satisfaction with nursing care provided by male nurses at the medical and surgical wards of Komfo Anokye Teaching Hospital [KATH].

**Methods:**

An inferential cross-sectional study design, in which the prevalence of a condition among an identified population is determined, was used. Using convenience sampling, 150 respondents who meet certain practical criteria and are available and willing to participate were sampled. Data from a context-based research instrument on the opinion, preference and satisfaction of patients with male nursing were analysed using *χ*^*2*^ test, Mann Whitney U test, ordinal logistic regression and logistic regression.

**Results:**

The study indicates that more females than males had ever been attended to by a male nurse for the period considered by the study, and females described male nurses as polite and courteous and were comfortable with their treatment. Being single [OR = 0.111, 95% CI (0.013–0.928)] and professing Islamic faiths [OR = 36.533, 95% CI (2.116–630.597)] were functions of respondents’ preference for a male nurse. Significantly too, affiliating to a religious sect (OR = 2.347, 95% CI [0.076–1.630]) and being educated (OR = 1.387, 95% CI [0.040–0.615]), were associated with higher odds of falling in one of the higher categories of satisfaction with nursing care provided by male nurses as against the lower categories.

**Conclusion:**

Although marital status, religious affiliation and educational level were the significant predictors of patients’ preference for, and satisfaction with care provided by male nurses, the effect of the other variables should not be overlooked. The finding disproves assertions on the negative effect of religion on male nurses. It is recommended that public awareness be created on the role of male nurses in the healthcare delivery system to promote acceptance of gender diversity in the nursing profession.

## Background

Nursing until the nineteenth century was not an activity thought to demand skill, training or commanded respect [1]. The profession was envisaged as a self-conscious occupation where anyone could freely describe themselves as ‘nurses’ and call what they did as nursing [2]. Gail (p.1), quoting Florence Nightingale intimated that: ‘nursing was left to those who were too old, too weak, too drunken, too dirty, too stupid or too bad to do anything else’ [1]. During the mid-nineteenth century, men were conceptualised as individuals whose thorny hands were detrimental to caring and as such, were classified unfit to be nurses [3]. Rather, the nursing profession was tagged with a feminine outlook due to Nightingale’s work during this same era [4]. Moreover, advances in medical techniques through the discovery and application of anaesthetics and antiseptic surgery [1, 5], coupled with the establishment of nursing training institutions during the middle and late nineteenth century produced a preponderance of females who were accepted as medical officers in various hospitals [6]. Currently, nursing continues to be largely a female dominated profession [7].

Even though the number of registered nurses and nursing training schools have increased globally, coupled with attempts made at increasing the number of individuals in the nursing profession [8], the percentage of men remains underrepresented [9, 10]. In the United States of America, the percentage of male nurses is an abysmally 10% [11] and just 1 % in Jamaica [12], though the inclusion and subsequent abundance of males in the nursing profession could contribute to augmenting nursing shortage and provide diversity in the profession [13]. Whereas some studies found evidence to suggest patients preference for male nurses [14], other influencing factors tend to perpetuate the myth that men are unsuitable to become nurses [13]: negative stereotypes, where reference is made to the nurse as “she” in textbooks and the subsequent omission of men from the history of nursing [15, 16], labelling of men in the nursing profession as “He-Man”, troublemaker, effeminate or gay [17, 18], the absence or lack of role models to offer advice about nursing for males [10, 19] and the absence of a male oriented approach to teaching in nursing schools [20]. Besides these factors, the cultural orientation of society about the unsuitability of males to provide care [21, 22] and the negative portrayal of male nurses by the media [18] tend to limit the presence of males in the nursing profession. These factors, in addition to the aforementioned, potentially impact the psychological orientation of male nurses who report high degrees of anxiety and tension on the job and are more likely to leave the profession early in comparison to their female counterparts [23–25]. Instances where these factors are absent, differential treatment are meted out to male nurses. Specifically, male nurses are marginalised, and prevented from performing certain personal and intimate care procedures such as electrocardiograms, catheterization and Papanicolaou smears, for female patients, as well as working in the labour and delivery units ([10, 20]-p. 61, [26, 27]).

In Ghana, nursing as a profession was introduced as a subset of colonialism and the introduction of Christianity in the nineteenth century [28]. The people of Ghana, then Gold Coast, had trouble accepting the profession especially on the part of women owing to cultural differences. Hence, boys who were school leavers, otherwise termed “bush-boys”, were the ones who underwent training as nurses [29]. However, as the years went by, women became more welcoming to the professions (nursing and midwifery) and therefore got trained [28]. Even though Ghana’s health sector is faced with serious shortage of human resources [30], the few available trained nurses, especially females, leave the country in droves in search of ‘greener pastures’ abroad, particularly the US and UK, with serious consequence on health delivery in Ghana [21].

At the Komfo Anokye Teaching Hospital, 1290 nurses were employed from 2007 to 2016. Out of this number, 1080 are women (84%) and 210 men. Currently in 2017, the nursing population of the hospital stands at 1577, with 1361 females (86%) and 216 males [31]. Whereas the female population increased by 26%, that of the male population increased by only 3% between 2016 and 2017. The gender imbalance that exist in nursing is a problem as it gives no regard to diversity [32, 33].

Though men in recent times have considered nursing as a preferred occupation for various reasons [34, 35], mixed feelings continue to persist about patients experiences on services provided by male nurses. Whereas in some studies, patients appraised their satisfaction with male nurses [14, 36, 37], others considered caring as an attribute of female nurses, which could imply a non-caring image of male nurses [38, 39]. Even within the same country [40, 41] and different countries [13, 37, 42], inconsistent findings have been reported on patient’s opinion and satisfaction with care provided by male nurses. The mixed reactions, and inconsistencies surrounding patients’ preference for, and satisfaction with care provided by male nurses [13, 14, 36–42], begs for further studies targeted at settling these differences. The presence of these inconsistencies continues to breed confounding evidence on patients’ preference for, general orientation towards, and acceptability of males, as nurses [9, 43, 44]. Amid all the inconsistencies witnessed in both developed and low-and middle-income countries, there is yet to be, a study on patient’s preference for, and satisfaction with nursing care by male nurses in Ghana though the percentage of male nurses channelled from the universities and nursing training colleges in the country keeps increasing. Primarily, this seed study sought to assess patients’ preference and satisfaction of care provided by male nurses at the Komfo Anokye Teaching Hospital, Ghana. The opinion of male and female patients regarding male nurses as caregivers is also reported.

### Theoretical framework

The underlying theory adapted for this study is the middle-range theory by Swanson [45]. In this theory, Swanson described the prevailing factors or actions which helps to foster positive patient outcomes [45, 46], restores [47] and unifies both patient and nurse [48] relationship. He defines care as “a nurturing way of relating to a value other toward whom one feels a personal sense of commitment and responsibility” [45]. This makes caring an integral component of nursing [49] due to the need for nurses to be self-knowledgeable and engrossed in ameliorating their patients’ discomfort [50, 51]. The middle-range theory is made up of five levels of care which helps to guide the actions of nurses, in which Level 1 examines the characteristics of caring persons; level II: commitment to providing caring; level III: the conditions that enhances or diminishes the likelihood of care being implemented by the interaction of the following variables (nurse, patient and organization); Level IV summarizes caring actions and level V are the consequences of caring [52]. This can be summarised as follows: knowing, being with, doing for, enabling and maintaining belief. The phenomena Kristen Swanson addressed reflects the process of nursing practice which could be best applied to this study where caring was the pivot upon which male and female nursing care processes were mirrored (viewed) in relation to patient’s satisfaction of care provided by male or female nurses. In holistic nursing care, the nurse must establish a rapport with the patient to enable her to get the patient’s cooperation. The nurse’s relationship with the patient provides basis for identifying the patient’s problems. The nurse through the caring process, plan with the patient and family, set short- and long-term goals to address the patient’s problem identified. Despite the use of these levels in previous nursing care studies, little attention was given to the incorporation of Level III in these studies [13]. This study builds on level III of the middle-range theory as used by Adeyemi-Adelanwa and colleagues [13] by assessing the factors which enhances or diminishes the likelihood of care provided by male nurses. Respondents demographic characteristics were used as predictors or independent factors for assessing their preference and satisfaction with male nursing care. It is important to indicate that preference and satisfaction are mutually inclusive and as such, using the same predictors has positive implication on the study findings. The theory’s effectiveness in strengthening nursing practice by improving patient satisfaction and outcome [46], and factors which are likely to diminish nursing care by male nurses in the future, made it useful for this study. Considering the negative label attributed to males in the nursing profession [17, 18] and the cultural orientation of society towards male nurses [21, 22], it is envisaged by this study that patients’ demographic characteristics could potentially facilitate or inhibit their preference for, and satisfaction with nursing care provided by male nurses.

## Methods

### Study context and design

The study was carried out at both the medical and surgical wards of the second largest hospital in Ghana, Komfo Anokye Teaching Hospital (KATH), Kumasi. KATH doubles as a major referral hospital serving mostly the regions in the northern part of Ghana [53]. The specific wards where the research was carried out were C1 (female ward), C2 (male ward), D3 (male ward) and D5 (female ward) of the surgical and medical wards respectively. C1 and C2 are on the first and second floor of the C-block respectively, whereas D3 and D5 are on the second and fourth floor of the D-block respectively. The blocks are linked by corridors. Each ward has a bed capacity of thirty-six (36). An inferential cross sectional study design, in which data were collected at a single point in time was used. The design preference was necessitated by the need to identify the prevalence of a condition, disease or a phenomenon among an identified population [54–56]. In this case, prevalence looks at the percentage of the population who prefer and are satisfied with nursing care provided by male nurses.

### Data and sample

A context based data on the opinion, preference and satisfaction of patients with nursing care provided by male nurses was used in this study. Data were collected within two weeks after the ethical approval on 20th March 2017. The underlying objective of the questionnaire [57] was to provide a breadth of understanding on patients’ estimation of the activities of male nurses within Ghana’s Health Sector, as well as provide a baseline information for prospective male nurses in Ghana. The need to identify respondents who meet certain practical criteria, are within accessible geographical proximity, and are available and willing to participate in the study, led to the use of convenience sampling [58, 59]. The sampling technique has been proven effective in health-related studies [60]. The technique is guided by the principle of homogeneity and as such, its results are not different from a study which made use of random sampling techniques [61]: it ensures that knowledge gained is representative of the population [62].

Going by this technique, 150 patients from the medical and surgical wards of KATH who were eighteen (18) years and above and had been admitted at least twenty-four (24) hours or more prior to the study were sampled for inclusion [63]. The draw back to this sampling technique is the presence of outliers and biases in the sampling procedure which could potentially undermine the representativeness of the study findings [64, 65]. This drawback was addressed by means of approaching all respondents who had been admitted in the aforementioned wards and only those who agreed to participate in the study were included. This approach was used as a criteria for checking biases and outliers since controlling all factors which could result in biases or outliers was neither possible in practice nor wise [66].

### Variables and measures

#### Satisfaction and preference for male nurse

Respondent’s satisfaction with male nursing care were assessed with the question: “indicate your satisfaction level on being cared for by a male nurse”, and were optioned as; “very unsatisfied (1), unsatisfied (2), satisfied (3), adequately satisfied (4), very satisfied (5)”. On the other hand, respondents’ preference for male nurse was assessed with the question: “Would you prefer to be attended to by a male nurse on any other visit to the hospital?” The options were; “Yes (0), No (1), Maybe (2)”. However, due to non-response for “maybe”, only “Yes/No” responses were used for the final analysis.

#### Opinion about nursing care provided by male nurses

Participants’ opinion about male nurses were assessed using Likert scaled responses ranging from very satisfied to very unsatisfied. Perception or opinion as used in this study reflects the reaction and disposition of patients during the care process. The variables included: politeness and courtesy of the male nurse, professional conduct of the male nurse, skilful discharge of duties by male nurse, male nurse creating a friendly atmosphere and answering all your questions, comfortable with receiving care from a male nurse and level of cooperation with the various duties carried out by the male nurse. The question preceding the perception questions was: “Have you ever been attended to by a male nurse during hospital visit in the last 3 months?”. This had a dichotomous response: (Yes/No).

#### Covariates

Respondents’ demographic characteristics which were used as covariates included; gender (male/female), age (18–24/25–34/35–44/45–54/55–64/65 and above), marital status (married/single/separated/divorced/widowed), occupation (public sector/self-employed/private sector/unemployed/student/retired), ethnicity (Akan/Ga-Adangbe/Guan/Ewe/Mole Dagbani), religious affiliation (Christianity/Islamic/traditionalist) and level of education (basic education/secondary/tertiary/none).

### Data management and analyses

Data were analysed using Predictive Analytics SoftWare (PASW) version 16. The study instrument established an alpha value of 0.834. Differences in frequencies among respondents’ demographic characteristics by gender were assessed using *χ*^*2*^ test analysis. Participants’ opinion about male nursing care were analysed using Mann Whitney U test due to the skewed distribution, and ordinal nature of the opinion parameters [67, 68]. We used ordinal logistic regression (OLR) to assess respondents’ satisfaction with male nursing care owing to the ordinal nature of the dependent variable. Respondent’s demographic characteristics was regressed upon their satisfaction with male nursing care, to calculate the crude odds ratio (OR). The good-of-test of the OLR was judged using Pearson’s, − 2 Log Likelihood Ratio and Test of parallel lines values. Respondent’s preference for male nurse was ascertained using logistic regression. Respondent’s basic information were all regressed upon participants’ preference for male nurse to calculate the crude OR. The statistical parameters; Hosmer-Lemeshow test and Nagelkerke R Square, were used to judge the model fit of the analysis. All statistical result were deemed significant at *p < .05*.

### Ethical consideration

Ethical clearance was sought from both the medical and surgical directorates of the Komfo Anokye Teaching Hospital and the ethical review committee of the School of Medical Sciences, KNUST on 20th March 2017, with identification number CHRPE/AP/181/17. Besides this, oral consent were sought from the study participants after presenting an introductory letter from the Department of Nursing, KNUST, which detailed the essence of the study. Only participants who agreed to participate in the study were included. Confidentiality and anonymity of response were strictly adhered to; no names were recorded on the questionnaires and there was no alteration of the information obtained.

## Results

### Respondents’ demographic characteristics

Respondents’ biodata by sex are presented in Table 1. Majority (108, 72%) of the respondents were below 45 years, married (58, 39%), self-employed (78, 52%) and had attained basic education (80, 53%). Overall, 86% of the study participants professed Christian beliefs and 72% were from the Akan ethnic group. Besides age, religious beliefs and ethnic groups, there was statistically significant differences between the male and female respondents based on the Pearson Chi-Squared test conducted. This proves the representativeness of the study sample (*p < 0.05*).

**Table 1:** Demographic characteristics of Respondents by sex

Variables	Gender			*p-value*
	Male	Female	Total	
	N (%)	N (%)	N (%)	
Age				
18–24	20 (20.8)	13 (24.1)	33 (22)	0.194
25–34	24 (25)	14 (25.9)	38 (25.3)	
35–44	28 (29.2)	9 (16.7)	37 (24.7)	
45–54	12 (12.5)	4 (7.4)	16 (10.7)	
55–64	6 (6.2)	9 (16.7)	15 (10)	
65 and above	6 (6.2)	5 (9.3)	11 (7.3)	
Marital Status				
Married	44 (45.8)	14 (25.9)	58 (38.7)	
Single	36 (37.5)	20 (37)	56 (37.3)	0.049^a^
Separated	6 (6.2)	8 (14.8)	14 (9.3)	
Divorced	6 (6.2)	7 (13)	13 (8.7)	
Widowed	4 (4.2)	5 (9.3)	9 (6)	
Occupation				
Public sector	12 (12.5)	6 (11.1)	18 (12)	
Self-employed	52 (54.2)	26 (48.1)	78 (52)	0.005^a^
Private sector	18 (18.7)	2 (3.7)	20 (13.3)	
Unemployed	2 (2.1)	8 (14.8)	10 (6.7)	
Student	6 (6.2)	7 (13)	13 (8.7)	
Retired	6 (6.2)	5 (9.3)	11 (7.3)	
Religious Beliefs				
Christianity	80 (83.3)	49 (90.7)	129 (86)	0.248
Islamic	12 (12.5)	5 (9.3)	17 (11.3)	
Traditionalist	4 (4.2)	0 (0)	4 (2.7)	
Educational level				
Basic education	52 (54.1)	28 (51.9)	80 (53.4)	0.015^a^
Secondary	26 (27.1)	7 (13)	33 (22)	
Tertiary	12 (12.5)	7 (13)	19 (12.7)	
None	6 (6.2)	12 (22.2)	18 (12)	
Ethnic Group				
Akan	64 (66.7)	44 (81.5)	108 (72)	
Ga-Adangbe	2 (2.1)	2 (3.7)	4 (2.7)	0.062
Guan	2 (2.1)	3 (5.6)	5 (3.3)	
Ewe	8 (8.3)	1 (1.8)	9 (6)	
Mole Dagbani	20 (20.8)	4 (7.4)	24 (16)	

^a^The Chi-square statistic is significant at the 0.05 level

### Patients’ opinion about nursing care provided by male nurses

Table 2 presents a Mann-Whitney output on patients’ perception about male nurses based on their gender. The mean ranks for each group indicates the perceptual difference between the male and female patients. Primarily, more females had been attended to by male nurses than their male counterparts during the hospital visits. The female patients had higher mean ranks than males in the following variables: male nurses as polite and courteous, skilful with their craft and provided a comforting atmosphere when taking care of their patients. However, the male patients scored higher mean ranks than the females regarding the professional conduct and cooperation with of male nurses. Despite these differences in mean score, just two opinion variables: politeness and courtesy and comfortability with male nurses, were statistically significant between the male and female patients.

**Table 2:** Perception about nursing care provided by male nurses

Variables	Gender	*N* = 150	Mean Rank	*p-value*
Attended to by a male nurse	Male	96	67.19	0.001^*^
	Female	54	90.28	
Professional about their duties	Male	96	75.75	0.916
	Female	54	75.06	
Cooperation with the duties of the male nurse	Male	96	75.65	0.944
	Female	54	75.24	
Polite and courteous	Male	96	70.16	0.019^*^
	Female	54	85.00	
Skilful manner	Male	96	73.79	0.480
	Female	54	78.54	
Friendly atmosphere	Male	96	75.31	0.935
	Female	54	75.83	
Comfortable	Male	96	70.42	0.029^*^
	Female	54	84.54	

^*^Statistically significant at *p* < 0.05

### Predictors of patients’ preference and satisfaction with nursing care provided by male nurses

From Table 3, two of participants’ demographic information; marital status and religious affiliation, were significant predictors of their preference for a male nurse. Particularly, those who were single had an 88.9% chance [OR = 0.111, 95% CI (0.013–0.928)] of preferring a male nurse on any other visit to the hospital as compared to the married patients. Also, respondents who professed Islamic faiths were about 3553% more likely to prefer a male nurse [OR = 36.533, 95% CI (2.116–630.597)] on any other visit as compared to the Christians. The predictive values explain roughly 44.3% of the variation in the dependent variable and the Hosmer-Lemeshow result proves fitness of the model (*p > 0.05*).

**Table 3:** Logistic regression analysis of patients’ preference with nursing care provided by male nurses

Would you prefer to be attended to by a male nurse on any other visit to the hospital (Yes/No)			
Covariates		OR	95% CI
Gender	Male	1	
	Female	0.306	0.046–2.039
Age	18–24	1	
	25–34	1.719	0.262–11.296
	35–44	0.104	0.010–1.046
	45–54	0.356	0.018–7.133
	55–64	0.337	0.008–14.147
	65 and above	1.505	0.000
Marital status	Married	1	
	Single	0.111	0.013–0.928*
	Separated	5.376	0.196–147.125
	Divorced	0.568	0.027–12.131
	Widowed	0.000	0.000
Occupation	Public sector	1	
	Self-employed	4.257	0.000
	Private sector	5.436	0.000
	Unemployed	8.180	0.000
	Student	2.767	0.000
	Retired	0.000	0.000
Ethnicity	Akan	1	
	Ga-Adangbe	0.000	0.000
	Guan	0.000	0.000
	Ewe	1.180	0.084–16.507
	Mole Dagbani	0.682	0.033–13.933
Religious beliefs	Christianity	1	
	Islamic	36.533	2.116–630.597*
	Traditionalist	0.000	0.000
Level of education	Basic education	1	
	Secondary	2.356	0.368–15.058
	Tertiary	0.857	0.087–8.395
	None	0.676	0.041–11.169

Nagelkerke R^2^: 0.443; Hosmer-Lemeshow Test: 0.889

*p < .05; 1: reference category

From Table 4, respondents’ religion and educational level were significant predictors of their satisfaction with nursing care provided by male nurses. Specifically, respondents who were affiliated to a religious sect (OR = 2.347, 95% CI [0.076–1.630]) and educated (OR = 1.387, 95% CI [0.040–0.615]), had higher odds of falling in one of the higher categories of satisfaction with nursing care provided by male nurses as against the lower categories.

**Table 4:** Ordinal logistic regression of patients’ satisfaction with nursing care provided by male nurses

Covariates	Model	
	OR	95% CI
Gender	1.099	−0.628 – 0.817
Age	0.856	−0.397 – 0.086
Marital status	1.123	−0.194 – 0.427
Occupation	1.015	−0.232 – 0.262
Ethnicity	0.988	−0.239 – 0.216
Religious beliefs	2.347	0.076–1.630*
Level of Education	1.387	0.040–0.615*
Goodness of fit test		
*Pearson*	0.000	
*-2 Log Likelihood Ratio*	257.043	
Test of parallel lines	1.000	

*p < 0.05

## Discussion

This is the first pilot study about patients’ preference and satisfaction with nursing care provided by male nurses in Ghana. Respondents’ preference for, and satisfaction with male nursing care were *functions* of their marital status, educational level and religious affiliation. Participants who were single and those who professed Islamic beliefs had lesser and higher odds, respectively, of preferring male nurses on any visit to the hospital. On the other hand, respondents’ religious affiliation and educational level were significant predictors of their satisfaction with male nursing care. In both cases, religious affiliation had a strong connotation on respondents’ preference and satisfaction with male nursing care. Despite the small representation of Islamic patients in comparison to the other religious faiths, our study finding defeats earlier assertions on the prejudice against male nurses by patients who professed Islamic beliefs [69]. This is an interesting finding and have positive effect on Ghana’s health sector considering the percentage of Islamic believers in the country and barriers to healthcare, especially among Muslim women in Ghana. For instance, most Muslim women shy away from maternal health services due to their religious obligation to maintain bodily sanctity and avoid exposing their body to male caregivers [70]. Thus, the positive acceptance of male nurses in our study could imply a positive outlook and professional conduct on the part of male nurses and the general acceptance of male nurses, cultural heterogeneity and the influence of the sampled wards in the study settings among the study participants. Perhaps, future qualitative studies from these sampled wards would be important to validate the quantitative studies through a nuanced presentation of patients’ subjective perception, preference and satisfaction with male nurses.

The fact that the other demographic characteristics, for instance age, had an insignificant effect on preference for, and satisfaction with male nurses is in good keeping for Ghana’s ageing population [71, 72], and the health of older adults [73, 74]. This development would help avert instances where older adults (patients) might refuse treatment from male nurses altogether. Though our study finding confirms previous studies where patients’ satisfaction with nursing care was irrespective of the nurses’ gender [75, 76], it contradicts other studies which reported a strong gender preference by female patients [77] and satisfaction with nursing care [78]. Patients general orientation with male nursing care was irrespective of whether the type of care provided were intimate care or not, as reported elsewhere [79, 80]. Most importantly, the significant findings on respondents’ satisfaction with male nursing care emphasises the criterion used to measure the quality of care provided by nurses [81]. This means that, some of the respondents positively appraised the quality of care provided by male nurses during hospital visits. Particularly, the influence of education and religious affiliation on respondents’ satisfaction with male nurses, connotes the positive influence of schooling and belonging to a religious sect on the behaviour and perception of individuals.

Besides this, majority of the participants’ in the respective medical and surgical units had been nursed by male nurses and opined that male nurses were polite, courteous and provided a welcoming atmosphere for them. In effect, though participants were rarely bothered about the gender of the nurses, they took cognizance of the attitude displayed by these nurses when they sought healthcare. Between the male and female divide, although more males than females participated in the study, statistically, more females had been attended to by male nurses, perceived, and described male nurses as polite and courteous and were comfortable with receiving care from a male nurse (Table 2). Even though men are not seen and described as natural caregivers, they seem to be doing well in the nursing profession considering the positive perception of patients towards them. Our study findings on respondent’s perception about male nurses is comparable with studies by Achora [82] who reported that male nurses are approachable, courteous and polite, and do create an environment conducive for their patient [13, 45].

Perhaps, the fact that male nurses are often stereotyped as “He-Man” and/or are “pressurized” by society due to the feminine orientation attached to nursing [15, 16], leaves a large room for male nurses to prove themselves as being worthy of their profession. Thus, their actions and activities during practice are key to their sustenance and continuance in the profession [24, 25, 34, 38, 39]. Relating this to Swanson’s [45] middle-range theory which describes nursing actions as key to aiding positive patient outcomes [45, 46], restores [47] and unifies both patient and nurse [48] outcomes, male nurses have a lot to give if the negative label they are often associated with is to be abated. Although male nurses were praised for their professionalism and thus, having larger proportions of them will boost Ghana’s health sector, the unfavourable view associated with males being in the nursing profession as described previously [15, 16], could potentially hinder their increased percentage in the profession [10, 19] just as witnessed by the KATH 2016/17 records [31].

There were no significant differences between patients’ gender, and the professional duties of male nurses, cooperating with male nurses, skilfulness of male nurses and the effectiveness of treatment received from a male nurse. Though some bottlenecks persist on patients’ opinion about nursing care by male nurses, the indicators used strongly point to the fact that with time, patients’ reception of male nurses will no longer be viewed with a gendered spectacle. Gradually, male nurses will be openly accepted by patients and will flourish in an environment where they will not be stressed and forced to resign faster than their female counterpart. This assertion is premised on the fact that negative perception of male nurses by patients eventually affects the care they receive from them [83] and affects nurse-patient relationship [84].

The strength of this study is that it pioneered a study on patients’ preference for and satisfaction with nursing care provided by male nurses in Ghana. Some limitations however are noted. Specifically, the study failed to examine patients’ opinion on specific intimate care services which they will or will not permit male nurses to perform on them. Given the fact that, religious affiliation, marital status and educational level significantly predicted patients’ satisfaction with male nurses, including such specifics (type of care) would have helped to reveal other salient patterns. Significantly too, though this study argued on the homogeneity of the study sample, as well as the generalisability of the study findings, it ought to be considered with caution due to the vulnerability of convenience sampling to some hidden biases.

## Conclusion

The study assessed a grey area on patients’ preference for and satisfaction with care from male nurses at the medical and surgical wards of the Komfo Anokye Teaching Hospital. Although marital status, religious affiliation and educational level, were just the significant predictors of their preference for, and satisfaction with male nursing care, the effect of the other variables should not be overlooked. The finding disproves assertions on the negative effect of religion on male nurses. It is recommended that professional socialization of male nurses be enhanced by developing role models and public awareness created on the role of male nurses in the healthcare delivery system to promote acceptance of gender diversity in the nursing profession. The pervading religious influence in the Ghanaian society, coupled with clarion calls and effort to improve the quality of education in Ghana, ought to be commended and given the necessary support to produce an enlightened and educated population. This is because, an enlightened and educated population would most likely view diversity in the nursing profession as a desirable objective since it increases affinity with the career: preference for nurses of the same gender by some patients.

## Data Availability

The datasets used and/or analysed during the current study are available from the corresponding author on reasonable request.

## Abbreviations

KATH: Komfo Anokye Teaching Hospital
KNUST: Kwame Nkrumah University of Science and Technology
PASW: Predictive Analytics SoftWare
